# Gene-based genome-wide association studies and meta-analyses of conotruncal heart defects

**DOI:** 10.1371/journal.pone.0219926

**Published:** 2019-07-17

**Authors:** Anshuman Sewda, A. J. Agopian, Elizabeth Goldmuntz, Hakon Hakonarson, Bernice E. Morrow, Deanne Taylor, Laura E. Mitchell, on behalf of the Pediatric Cardiac Genomics Consortium

**Affiliations:** 1 Department of Epidemiology, Human Genetics and Environmental Sciences and Human Genetics Center, UTHealth School of Public Health, Houston, Texas, United States of America; 2 Department of Pediatrics, University of Pennsylvania Perelman School of Medicine, Philadelphia, Pennsylvania, United States of America; 3 Division of Cardiology, The Children’s Hospital of Philadelphia, Philadelphia, Pennsylvania, United States of America; 4 Center for Applied Genomics, The Children’s Hospital of Philadelphia, Philadelphia, Pennsylvania, United States of America; 5 Department of Genetics, Albert Einstein College of Medicine, Bronx, New York, United States of America; 6 Department of Biomedical and Health Informatics, The Children's Hospital of Philadelphia, Philadelphia, Pennsylvania, United States of America; University of Sydney, AUSTRALIA

## Abstract

Conotruncal heart defects (CTDs) are among the most common and severe groups of congenital heart defects. Despite evidence of an inherited genetic contribution to CTDs, little is known about the specific genes that contribute to the development of CTDs. We performed gene-based genome-wide analyses using microarray-genotyped and imputed common and rare variants data from two large studies of CTDs in the United States. We performed two case-parent trio analyses (N = 640 and 317 trios), using an extension of the family-based multi-marker association test, and two case-control analyses (N = 482 and 406 patients and comparable numbers of controls), using a sequence kernel association test. We also undertook two meta-analyses to combine the results from the analyses that used the same approach (i.e. family-based or case-control). To our knowledge, these analyses are the first reported gene-based, genome-wide association studies of CTDs. Based on our findings, we propose eight CTD candidate genes (*ARF5*, *EIF4E*, *KPNA1*, *MAP4K3*, *MBNL1*, *NCAPG*, *NDFUS1* and *PSMG3*). Four of these genes (*ARF5*, *KPNA1*, *NDUFS1* and *PSMG3*) have not been previously associated with normal or abnormal heart development. In addition, our analyses provide additional evidence that genes involved in chromatin-modification and in ribonucleic acid splicing are associated with congenital heart defects.

## Introduction

Congenital heart defects (CHDs) are the most common group of birth defects, with a prevalence of approximately 1% in live births [1]. CHDs are also the leading cause of birth defect related mortality [2] and account for the largest percentage of birth defect associated hospitalizations and hospitalization-associated costs [3]. In the United States, it is estimated that there are approximately 2.4 million CHD survivors (1.4 million adults, 1 million children) [4], the majority of whom will require lifelong cardiac care. Despite the impact on affected patients, their families, and the healthcare system, the causes of CHDs are not well defined [5].

There are many different CHD phenotypes, of which approximately one-third involve the cardiac outflow tracts and great arteries [6]–structures that develop from the cardiac neural crest and secondary heart field [7]. This subgroup of CHDs, collectively referred to as conotruncal heart defects (CTDs), includes some of the most severe and costly birth defects [3, 8]. In addition to their shared embryologic and anatomic basis, there is substantial evidence that the various CTD phenotypes (e.g. tetralogy of Fallot (TOF), truncus arteriosus) share common genetic underpinnings. For example, nationwide, population-based studies conducted in Norway and Denmark indicate that CTDs aggregate within families (recurrence risk ratios for CTDs in first-degree relatives: 9–12) [9, 10] and that affected relatives of patients with a CTD are at a higher relative risk for CTDs (sibling CTD recurrence risk ratio: 9.0, 95% confidence interval (CI) 4.0–20.0) than for other types of CHDs (sibling non-CTD, CHD recurrence risk ratio: 3.6, 95% CI 2.4–5.5) [9]. Further, there is evidence that, within affected relative-pairs, the specific type of CTD can differ. For example, among 28 CTD-affected siblings of patients with TOF, 17 also had TOF whereas 11 had a different CTD phenotype [11]. Additional evidence that the various CTD phenotypes share a common genetic basis is provided by the phenotypic characteristics of defined genetic syndromes. For example, in patients with the 22q11.2 deletion syndrome, the most common cardiac defects are CTDs, but the specific CTD phenotype (e.g. TOF, interrupted aortic arc) varies across patients with this deletion [12].

Studies of syndromes that include CTDs, such as the 22q11 deletion syndrome, have provided some clues regarding the specific genes that may be involved in determining the risk of CTDs (e.g. *TBX1* [13]). In addition, studies of rare, presumably pathogenic, copy number variants [14–16], and inherited [17] and *de novo* [17, 18] single nucleotide variants have identified genes that may contribute to the risk of CTDs [18, 19]. Yet, most affected patients do not carry a confirmed or suspected rare, causative variant. Moreover, rare variants, in particular rare *de novo* variants, do not account for the observed increase in risk of CTDs among the relatives of affected patients.

Since rare, pathogenic variants are unlikely to fully account for the population prevalence or familial recurrence of CTDs, additional genetic mechanisms must also contribute to disease risk. While the involvement of more common variants that have more moderate impacts on CTD risk seems likely, genome-wide association studies (GWAS) [20–23] of common single nucleotide polymorphisms (SNPs) have identified only two genome-wide significant associations for CTDs (rs11065987, p = 7.7E-11 and rs7982677, p = 3.03E-11) [22]. However, given the huge number of variants evaluated in GWAS, the threshold for statistical significance is quite stringent (i.e. p<5E-08). Consequently, the lack of significant findings for CTDs may well reflect low study power rather than the lack of common, CTD-related genetic variants.

Gene-based GWAS provide an additional strategy for identifying disease-related genes, but, to our knowledge, there are no published gene-based GWAS for CTDs. Compared to SNP-based GWAS, gene-based studies have the advantage of a less stringent threshold for statistical significance (e.g. Bonferroni corrected p-value for 20,000 genes, 2.5E-06). In addition, SNP-based analyses generally exclude rare variants, due to low statistical power [24], whereas gene-based analyses can incorporate data from both common and rare variants [25] and, therefore, capture more genomic variation than SNP-based analyses. Given these advantages, we have undertaken gene-based analyses and meta-analyses using data from several large CTD datasets.

## Materials and methods

### Study subjects

#### The Children's Hospital of Philadelphia (CHOP)

Informed consent was obtained under a protocol approved by the Institutional Review Board for the protection of human subjects at CHOP. Adult subjects (parents or guardians) provided written consent for themselves and their minor children. Patients diagnosed with CTDs and their available parents of all races and ethnicities were recruited through the Cardiac Center at CHOP from 1992–2010 [21].

Patients with the following diagnoses were included in the study: TOF, persistent truncus arteriosus, D-transposition of great arteries (TGA), double outlet right ventricle, ventricular septal defects (conoventricular, posterior malalignment and conoseptal hypoplasia types), aortic-pulmonary window, interrupted aortic arch and isolated aortic arch anomalies. Cardiac diagnoses were confirmed using medical and operative reports as well as imaging (e.g., echocardiography, cardiac magnetic resonance imaging, cardiac catheterization) records. All potential patients were tested for the 22q11.2 deletion syndrome using fluorescence *in situ* hybridization and/or multiplex ligation-dependent probe amplification using standard techniques. Patients with a confirmed 22q11.2 deletion were excluded [26]. Patients with a clinically diagnosed chromosomal abnormality, single gene mutation, teratogenic syndrome or known maternal risk factor (e.g. diabetes, anticonvulsant use) were also excluded [21].

The CHOP patients were previously microarray genotyped in two phases. In the first phase, cases with any CTD phenotype and of any race and ethnicity, and their parents were genotyped to generate data for a case-parent trio study. In the second phase, only non-Hispanic Caucasian cases (based on self- or parental-reported race/ethnicity) were genotyped to generate data for a case-control study. Control data were obtained from existing microarray genotyped data from pediatric controls that were recruited during well child visits at CHOP [27].

#### Pediatric Cardiac Genomics Consortium (PCGC)

Informed consent was obtained from each participating individual or their parent or guardian in accordance with protocols approved by the Institutional Review Board of each participating institution. Patients with a CHD and their available parents of all races and ethnicities were recruited as part of the PCGC Congenital Heart Defect GEnetic NEtwork Study from 2010–2012 [18, 28, 29]. PCGC recruitment took place at five main clinical sites (including CHOP) and four satellite clinics. The patients recruited by PCGC through CHOP do not overlap with the CHOP patients described above. Participant information was collected through medical records, electronic case reports, and personal interviews. Our studies were restricted to include patients with a CTD (as described above) and without a clinically diagnosed chromosomal or genetic disorder.

### Genetic methods

Blood samples were collected from each patient and pediatric control. When blood collection was scheduled in conjunction with a surgical procedure, the sample was collected prior to any blood transfusion. Blood or saliva samples were collected from available parents of patients. DNA extraction was performed using standard techniques.

Genome-wide microarray genotyping was performed at the CHOP Center for Applied Genomics. Samples collected at CHOP were genotyped using Illumina HumanOmni-2.5 or Illumina HumanHap550 (v2, v3), or 610 BeadChip platforms. Samples collected as part of the PCGC (including the PCGC samples collected at CHOP) were genotyped on the Illumina HumanOmni-1 or HumanOmni-2.5 platforms. Additional details regarding the CHOP and PCGC samples are provided elsewhere [18, 21, 27, 28].

### Imputation and quality control (QC) procedures

The microarray genotyped data from CHOP and PCGC were imputed using Impute2 v2.3.0 and pre-phased haplotype data obtained from the 1000 Genomes Project (Phase-I integrated v3 variants set) as the reference [30]. Due to differences in the genotyping platforms, the CHOP and PCGC cohorts were imputed separately.

Standard QC procedures were performed for each dataset using PLINKv1.07 before and after imputation [31]. Before imputation, the array data were checked for strand and coding errors. Trios were removed if more than 1% of genotyped SNPs had Mendelian errors. Suspected duplicate samples were identified using pairwise identify-by-descent estimation and samples with pi-hat greater than 0.6 were removed. Samples with genotyping rates less than 95% were also removed. In addition, variants with minor allele frequency (MAF) less than 1%, genotyping rates less than 90%, or deviation from Hardy Weinberg Equilibrium (HWE) in controls (p<1E-05) were excluded, as were all non-autosomal variants.

After the pre-imputation exclusions, the CHOP data from different platforms (HumanOmni-2.5, HumanHap550K v2, 550K v3 and 610K) were combined and only those variants present on all platforms (N = 283,977 SNPs) were used for imputation. Similarly, the PCGC data from different Illumina platforms (HumanOmni-1 and HumanOmni-2.5) were combined and only those SNPs present on both platforms (N = 624,419 SNPs) were used for imputation. For each dataset, haplotypes were pre-phased using SHAPEIT2 v2.727 [32] and imputation was performed using Impute2 v2.3.0 [30]. A genotype was imputed only if the posterior probability value exceeded 0.9, the default calling threshold for Impute2. After imputation, we excluded variants with poor imputation quality (Impute2 information metric score <0.8), or genotyping rates less than 90%. Samples with genotyping rates less than 95% and all insertions or deletions were removed. For all case-control comparisons, variants were evaluated for deviation from HWE in the pediatric control group using the exact test [33] implemented in PLINK, and variants with p<1E-05 were excluded. Because we were interested in assessing both rare and common variants, the post-imputation QC procedures did not include restrictions based on MAFs.

### Statistical analysis

Genome-wide gene-based analyses were conducted, as described below. Because the various gene-based approaches have different underlying assumptions, strengths and limitations, we used two different gene-based approaches, eFBAT-MM and SKAT-C, to optimize the probability of identifying CTD-related genes. All analyses included all autosomal RefSeq genes, defined by the transcription start-stop coordinates (Genome Reference Consortium Human genome build 37 or hg19 reference assembly) in the RefSeq gene records and we included variants that were 1kb upstream or downstream of each gene.

#### Family-based analyses

Data for case-parent trios ascertained through CHOP (CHOP-Trios) and PCGC (PCGC-Trios) trios were analyzed separately using an extension of the family-based multi-marker association test (eFBAT-MM) [34]. This test (i.e. eFBAT-MM) is a burden-type approach that collapses variant-level statistics over a gene or region to obtain a single p-value and makes the assumption that all associated variants in the gene or genetic region affect the phenotype in the same direction. The variants were weighted by the inverse of the MAF estimated from the parental genotypes. Meta-analysis of the gene p-values from the CHOP and PCGC trios was performed using Fisher’s combination of probability method [35].

#### Case-control analyses

For the present study, we formed two independent, case-control (CC) datasets using the microarray genotyped and imputed data from CHOP. The first dataset included the Caucasian subset of patients from the CHOP trios and an equal number of Caucasian pediatric controls (CHOP-CC1). The second dataset included a second set of Caucasian patients with a CTD and an equal number of Caucasian pediatric controls (CHOP-CC2). There was no overlap in the cases or the controls included in CHOP-CC1 and CHOP-CC2.

The two CHOP case-control datasets (CHOP-CC1 and CHOP-CC2) were analyzed separately using the sequence kernel association test for the combined effect of common and rare variants (SKAT-C) [36]. Using this approach, separate scores were calculated for rare and common SNPs and these scores were combined as a weighted sum to calculate the gene p-value. The SKAT-C recommended default parameters were used for variant weighting and analysis. To control for population stratification bias, only non-Hispanic Caucasian cases (based on self- or parental report) were included in the analyses. Since race and ethnicity were based on self-report (rather than ancestry informative genetic markers), each analysis was also adjusted for the first genotypic principal component. Genotypic principal component analyses were conducted in Golden Helix SVS8.1, using the default parameter settings (MAF-based allele classification, additive genetic model and data for each marker were normalized by its theoretical standard deviation under HWE) (Golden Helix, Inc., Bozeman, MT, www.goldenhelix.com). Meta-analysis of the gene p-values from the two case-control series was performed using Fisher’s combination of probability method [35]. Meta-analyses combining results from the eFBAT-MM and SKAT-C analyses were not performed, given the overlap in patients (i.e. the non-Hispanic Caucasian cases in the CHOP-Trios are the case group for CHOP-CC1) and the differences in the assumptions underlying the two analytic approaches.

For each of the family-based and case-control analyses, the genomic inflation factor (λ) was calculated (for the case-control analyses, λwas calculated using values that adjusted for the first genotypic principal component) and a quantile-quantile (Q-Q) plot was constructed to check for deviation of the genome-wide observed distribution of the test statistic from the expected null distribution. Genes with association p-values less than the Bonferroni-corrected p-value (based on the number of genes in each analysis) were considered genome-wide significant. Genes with p-values greater than the Bonferroni-corrected p-values but less than 1E-03 were considered to be suggestive of an association.

#### Gene-set enrichment analysis

Genes with p<0.01 in the eFBAT-MM or SKAT-C meta-analyses were evaluated together for gene-annotation enrichment using MetaCore^TM^ (Thomson Reuters, Life Science Research, https://portal.genego.com/metacore). A false-discovery rate (FDR) corrected p-value less than 0.05 was used to identify significant pathway maps and Gene Ontology (GO) processes. REVIGO was used for clustering GO terms based on p-values and semantic similarity score (simRel) [37]. The simRel scores range from 0 to 1 and we used a score threshold of 0.4 for filtering GO terms.

#### Gene annotation and prioritization

To prioritize genes with at least suggestive evidence of association with CTD (p<1E-3, in either the family-based or case-control meta-analysis), for future investigations, we considered: (1) whether the meta-analysis p-value for the gene was lower than the p-values in contributing datasets i.e. the evidence for association was stronger in the combined data than in either of the individual datasets; and, (2) gene expression levels, based on heart expression data from E9.5 and E14.5 mouse embryos [18]. For each gene with a meta-analysis p-value lower than the p-values for the contributing datasets, we annotated the variants that were included in our analyses, for location, function, MAF in the genome aggregation database [38], Combined Annotation Dependent Depletion (CADD) phred-scaled scores [39], Genome-Wide Annotation of VAriants (GWAVA) [40] and Genomic Evolutionary Rate Profiling scores [41]. Genes with meta-analysis p-values lower than the p-values from the contributing datasets, and with heart expression data in the top quartile at E9.5 or E14.5 were considered strong candidates for future investigations.

## Results

**Fig 1:**
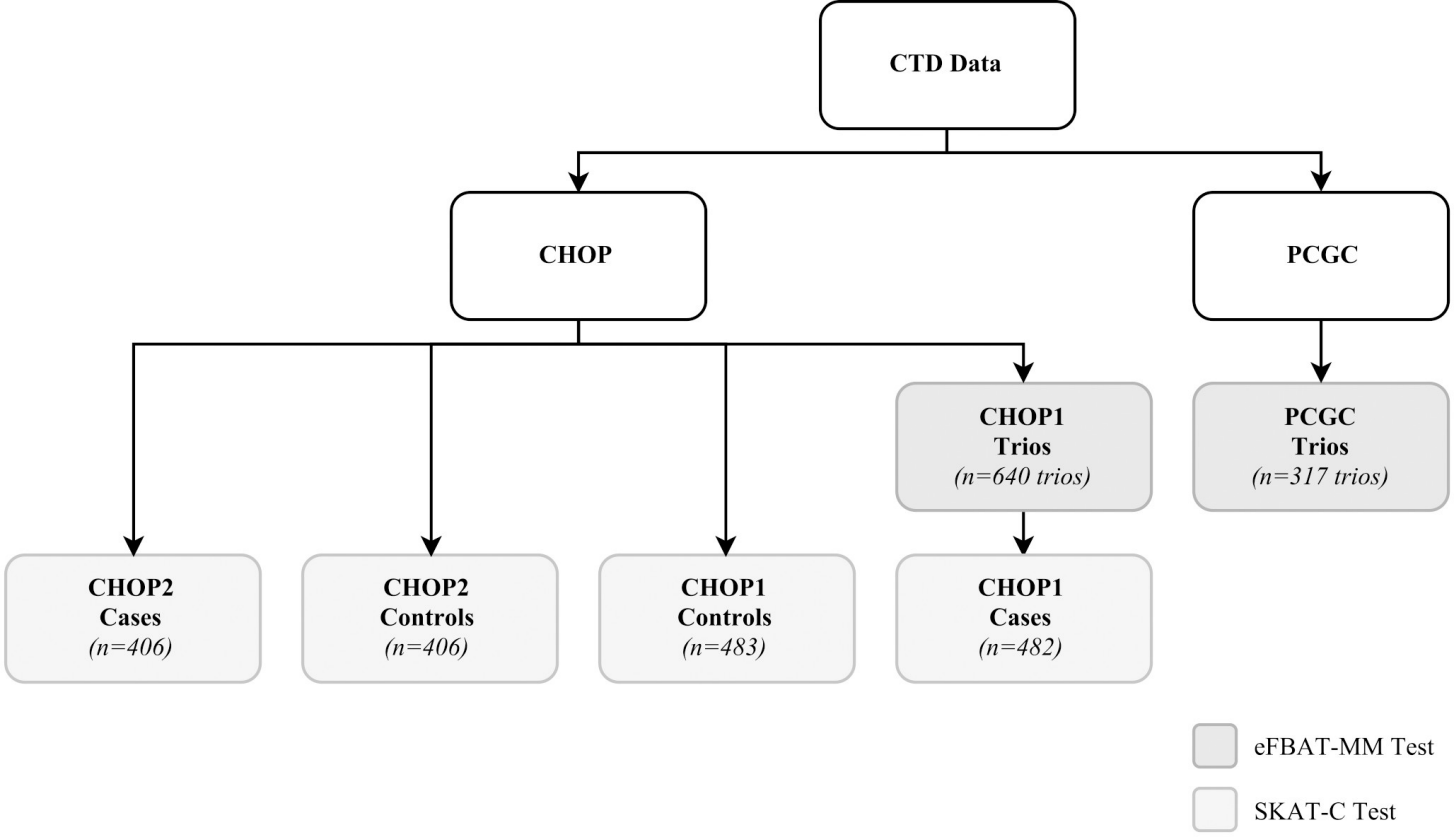
Summary of conotruncal heart defects data cohorts. The participants were recruited from the Children’s Hospital of Philadelphia (CHOP) and the Pediatric Cardiac Genomics Consortium (PCGC). CHOP-Trios and PCGC-Trios were analyzed using eFBAT-MM whereas SKAT-C was used to analyze the two case-control cohorts (CHOP-CC1 and CHOP-CC2). The cases in CHOP-CC1 are the Caucasian subset of cases in CHOP-Trios. One of 483 Caucasian cases was excluded during QC procedures prior to SKAT-C analysis.

After QC exclusions, there were 640 CHOP trios and 317 PCGC trios for family-based analyses (Fig 1). In addition, there were 482 patients with CTD and 483 controls for CHOP-CC1, and 406 patients with CTD and 406 controls for CHOP-CC2. In both sets of trios, patients were predominantly Caucasian (Table 1). The two case-control datasets were restricted to Caucasians participants. In all groups, the most common heart defect was TOF.

**Table 1:** Characteristics of patients with conotruncal defects in the Children’s Hospital of Philadelpia (CHOP) and Pediatric Cardiac Genomics Consortium (PCGC) datasets.

	N (%)					
	CHOP^-^Trios / CHOP-CC1^a^ (n = 640)		CHOP-CC2 (n = 406)		PCGC-Trios (n = 317)	
**Race/ethnicity**						
**Non-Hispanic Caucasian**	483	(75.5)	406	(100.0)	244	(70.1)
**Other**	157	(24.5)	0	(0.0)	73	(29.9)
**Sex**						
**Male**	387	(60.5)	236	(58.1)	192	(60.6)
**Female**	253	(39.5)	170	(41.9)	125	(39.4)
**Conotruncal defect phenotype**						
**Tetralogy of Fallot**	250	(39.1)	134	(33.0)	104	(32.8)
**D-transposition of the great arteries**	125	(19.5)	80	(19.7)	64	(20.2)
**Ventricular septal defects**	133	(20.8)	109	(26.8)	44	(13.9)
**Double outlet right ventricle**	66	(10.3)	25	(6.2)	46	(14.5)
**Isolated aortic arch anomalies**	30	(4.7)	22	(5.4)	7	(2.2)
**Persistent truncus arteriosus**	18	(2.8)	19	(4.7)	13	(4.1)
**Interrupted aortic arch**	11	(1.7)	10	(2.5)	9	(2.8)
**Other**	7	(1.1)	7	(1.7)	30	(9.5)

^a^The cases used in CHOP-CC1 are the subset of the cases included in the CHOP-Trios (i.e. the non-Hispanic Caucasian cases, N = 483).

### Gene-based GWAS of individual datasets

The number of variants and genes included in each analysis are summarized in Table 2. The genotype concordance for the imputation was >90%. The Q-Q plots (S1–S4 Figs) and genomic inflation factors (Table 2) provided little evidence for systematic bias in the observed p-values. No genome-wide significant associations were identified in the analyses of the individual datasets. The number of genes with suggestive evidence of association (p<1E-03) ranged from 13 to 27 (Table 2). There was no overlap across datasets or analyses with respect to the genes with suggestive evidence of association. Detailed genome-wide results for each analysis are included in Tables B-E in S1 File.

**Table 2:** Summary of eFBAT-MM and SKAT-C analyses and results.

	eFBAT-MM		SKAT-C	
	CHOP-Trios	PCGC Trios	CHOP-CC1	CHOP-CC2
**Total variants**	5,578,860	6,812,971	5,601,587	5,601,152
**Rare variants^a^**	3,446,735	4,502,285	3,502,419	3,495,988
**Number of genes**	21,256	22,247	21,212	21,269
**Genomic inflation factor (λ)**	1.03	1.04	1.09	1.09
**Genes with p<1E-03^b^**	13	13	25	27
	*CBLN2* *C22orf39* *NCOA2* *CEP95* *DDX5* *SQRDL* *SLMO2-ATP5E* *TRIP13* *PADI3* *RBM47* *PRR14* *ZC3H18* *CREBZF*	*MIR518C* *GOLGA2P9* *POU6F2* *LCE4A* *TMEM206* *LOC100996349* *ASAH2* *MBNL1* *IGFBPL1* *RNF44* *IRAK2* *DDX59* *ADGRA3*	*FAM225A* *ABCB4* *STK33* *BLOC1S6* *MIR3916* *LEXM* *DACT3* *MIR99AHG* *TRIP10* *LINC00620* *DACT3-AS1* *NPPC* *GPATCH1* *AVPR1A* *FLNC* *NCAPG* *SHD* *ARAP3* *FIGN* *NCAN* *SYMPK* *MIR548AA2* *MIR548D2* *BHMG1* *FCGR3B*	*PSMG3* *NEXN* *FUBP1* *PSMG3-AS1* *DNAJB4* *TSSC4* *TSPAN10* *MKX-AS1* *MKX* *UHMK1* *ATG9B* *MMP19* *CRTAM* *NPLOC4* *GRID2* *COPZ2* *PTPRT* *PYGL* *COA5* *EEF1B2* *APOPT1* *GATC* *ACTL7B* *NDUFS1* *LOC101927653* *AACS* *EPHB4*

^a^ Variants with minor allele frequency <0.05

^b^ Genes are listed by p-value (lowest to highest). Specific p-values are provided in Tables B-E in S1 File.

### Meta-analysis

**Fig 2:**
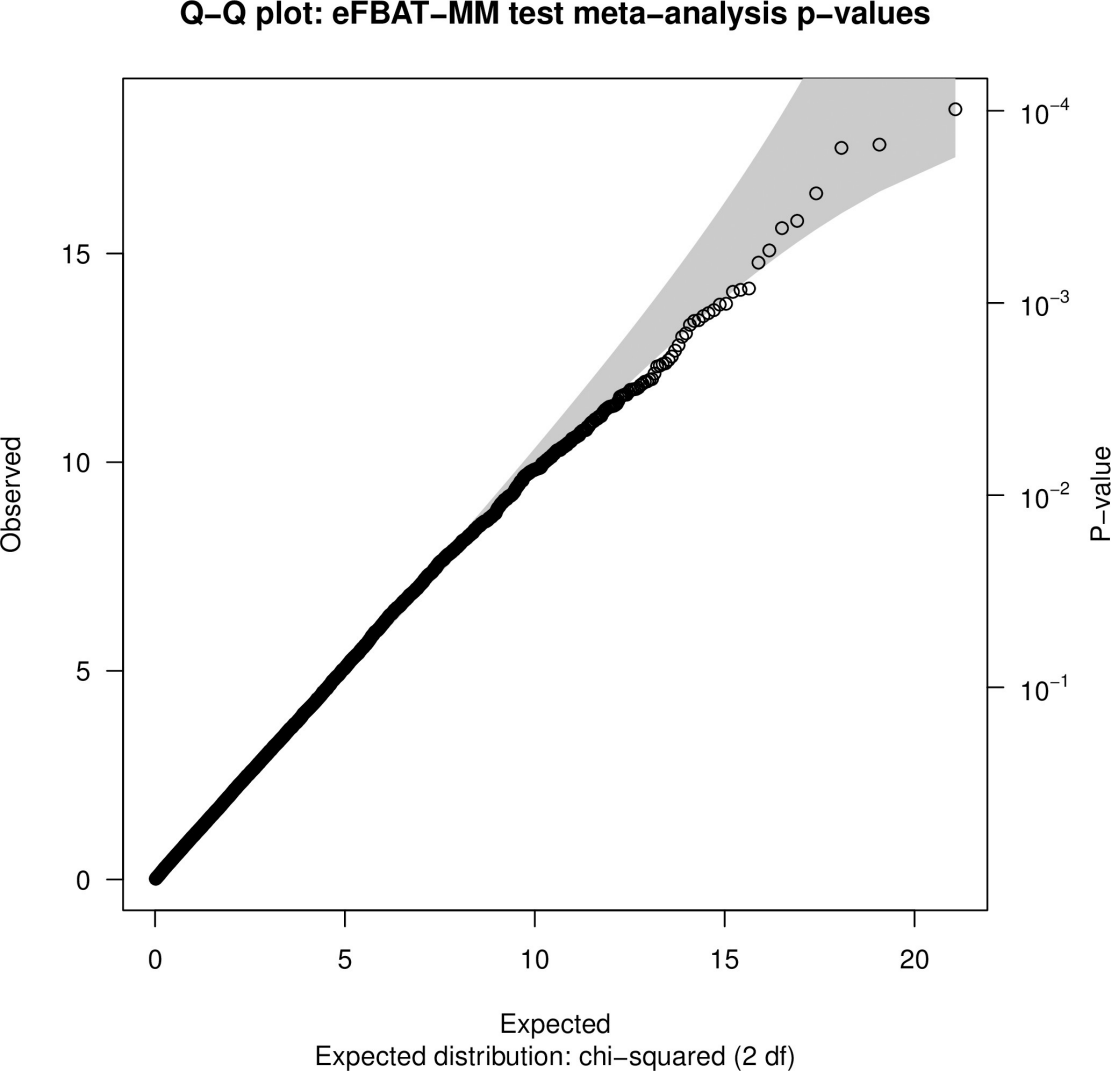
Quantile-quantile plot of eFBAT-MM test gene-level meta-analysis p-values.

**Fig 3:**
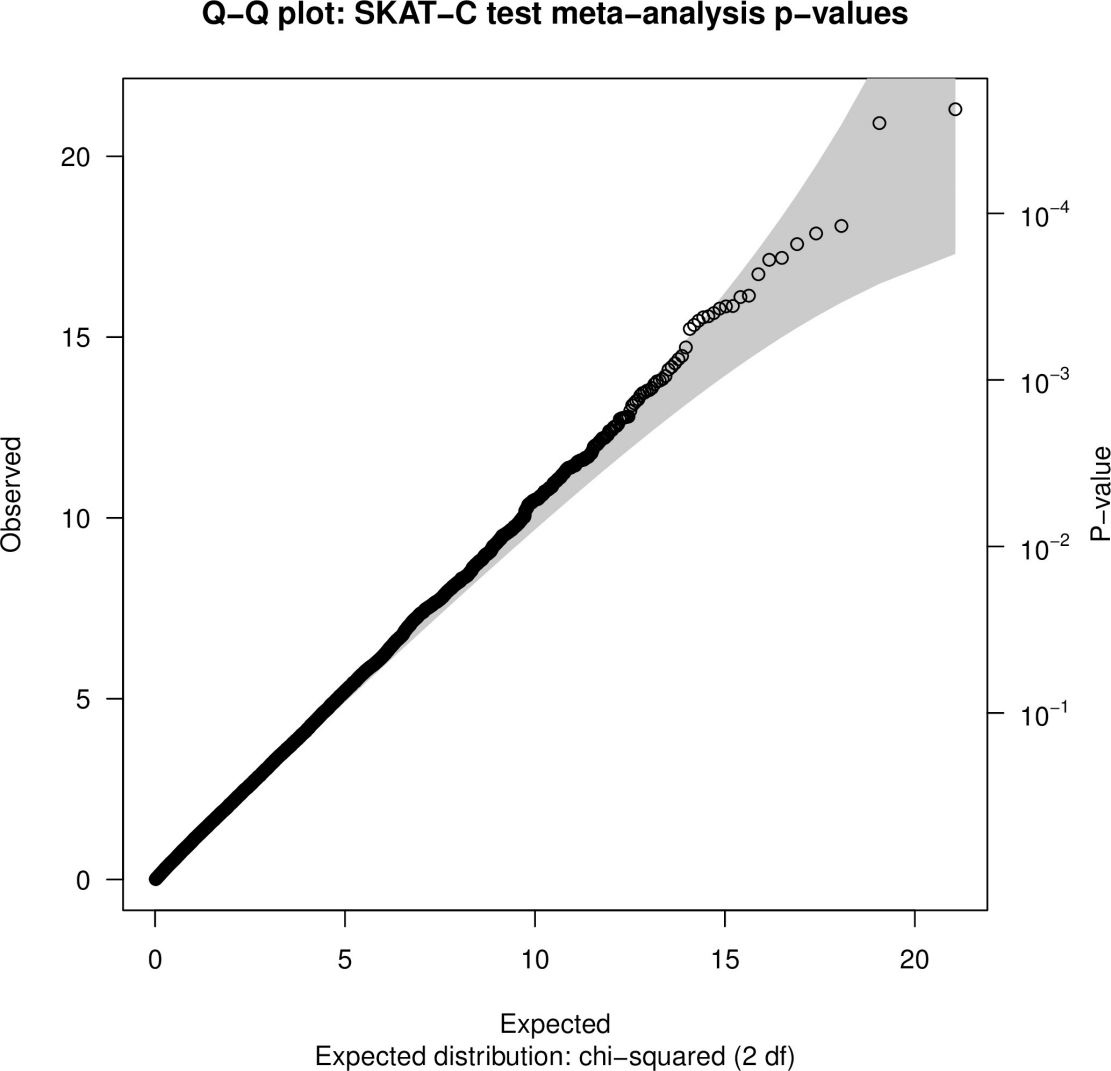
Quantile-quantile plot of SKAT-C test gene-level meta-analysis p-values.

Separate meta-analyses were conducted using eFBAT-MM p-values for 21,170 genes that were analyzed in both the CHOP and PCGC trios, and from SKAT-C p-values for 21,077 genes that were analyzed in both the CHOP-CC1 and CHOP-CC2 case-control studies. The Q-Q plots (Figs 2 and 3) and genomic inflation factors provided little evidence for systematic bias in the observed p-values. No gene achieved genome-wide significance in either meta-analysis (Table F in S1 File provides p-values for all genes assessed in each meta-analysis). Suggestive evidence of association (p<1E-03) was obtained for 11 genes (8 protein coding, 2 pseudogenes, 1 RNA gene) in the trio-based meta-analysis (Table 3) and for 27 genes (23 protein coding, 4 RNA genes) in the case-control based meta-analysis (Table 4).

**Table 3:** Genes with suggestive evidence of association (p<1E-03) in the trio-based meta-analysis.

		CHOP-Trios (640 trios)		PCGC-Trios (317 trios)		Meta-analysis
Gene	Function	Number of variants	p-value^a^	Number of variants	p-value^a^	p-value
***POU6F2***	Protein coding	2,662	8.9E-02	2,738	8.7E-05	9.8E-05
***MBNL1***	Protein coding	534	3.3E-02	789	3.7E-04	1.5E-04
***SIGLEC11***	Protein coding	37	4.1E-03	53	3.1E-03	1.6E-04
***GOLGA2P9***	Pseudogene	38	9.8E-01	83	2.3E-05	2.7E-04
***MAP4K3***	Protein coding	1,020	1.6E-03	1,063	2.1E-02	3.7E-04
***CBLN2***	Protein coding	39	2.4E-04	52	1.5E-01	4.1E-04
***ROR1***	Protein coding	1,585	2.4E-02	1,954	2.0E-03	5.3E-04
***LINC00207***	RNA gene	26	2.0E-02	25	2.9E-03	6.2E-04
***KPNA1***	Protein coding	317	5.4E-02	352	1.5E-03	8.4E-04
***GLIPR1***	Protein coding	81	2.9E-02	104	2.9E-03	8.6E-04
***LOC100996349***	Pseudogene	7	4.2E-01	15	2.0E-04	8.8E-04

^a^ gene-level p-values from eFBAT-MM test

**Table 4:** Genes with suggestive evidence of association (p<1E-03) in the case-control based meta-analysis.

		CHOP-CC1 (482 CTD patients/483 controls)		CHOP-CC2 (406 CTD patients/ 406 controls)		Meta-analysis
Gene	Function	Number of variants	p-value^a^	Number of variants	p-value^a^	p-value
***PSMG3***	Protein coding	41	6.5E-02	40	2.6E-05	2.4E-05
***FAM225A***	RNA gene	6	3.9E-05	7	5.3E-02	2.9E-05
***ABCB4***	Protein coding	252	9.9E-05	272	9.5E-02	1.2E-04
***LEXM***	Protein coding	208	2.5E-04	185	4.2E-02	1.3E-04
***SHD***	Protein coding	28	6.9E-04	31	1.8E-02	1.5E-04
***NEXN***	Protein coding	150	5.1 E-01	162	3.0E-05	1.9E-04
***LOC100287036***	Protein coding	12	6.3E-03	15	2.5E-03	1.9E-04
***PSMG3-AS1***	RNA gene	29	1.8 E-01	28	1.1E-04	2.3E-04
***PRKD2***	Protein coding	59	1.2E-03	67	2.3E-02	3.1E-04
***DACT3***	Protein coding	16	3.1E-04	14	9.1E-02	3.2E-04
***EIF4E***	Protein coding	237	2.6E-02	271	1.2E-03	3.6E-04
***MKX-AS1***	RNA gene	66	1.8 E-01	80	1.8E-04	3.6E-04
***MKX***	Protein coding	323	1.5 E-01	406	2.2E-04	3.7E-04
***RGS16***	Protein coding	11	5.3E-03	11	6.7E-03	4.0E-04
***CHODL***	Protein coding	1,101	2.8E-02	1,396	1.3E-03	4.2E-04
***NCAPG***	Protein coding	76	6.8E-04	84	5.5E-02	4.2E-04
***DNAJB4***	Protein coding	43	3.3 E-01	44	1.2E-04	4.4E-04
***FUBP1***	Protein coding	79	8.1 E-01	91	5.2E-05	4.7E-04
***DACT3-AS1***	RNA gene	14	4.7E-04	12	9.5E-02	4.9E-04
***STK33***	Protein coding	769	1.1E-04	784	5.7 E-01	6.4E-04
***DCAF16***	Protein coding	22	1.7E-03	28	4.0E-02	7.2E-04
***ACTL7B***	Protein coding	21	9.5E-02	20	7.5E-04	7.5E-04
***NDUFS1***	Protein coding	181	8.6E-02	192	8.8E-04	8.0E-04
***FBXO47***	Protein coding	66	2.2E-03	52	3.7E-02	8.4E-04
***ARF5***	Protein coding	25	3.8E-03	24	2.2E-02	8.7E-04
***PYGL***	Protein coding	114	1.7 E-01	112	5.4E-04	9.5E-04
***SYMPK***	Protein coding	234	7.8E-04	251	1.2 E-01	9.9E-04

^a^ gene-level p-values from SKAT-C

We identified genes that have previously been implicated in heart development and structural heart malformations (e.g. *MBNL1*, *ROR1*), and known disease-related genes (e.g. *NEXN*, dilated cardiomyopathy; *NDUFS1*, mitochondrial complex I deficiency). Several of the identified genes are also annotated to biological processes that are important during embryonic heart development including transcription (*FUBP1*, *POU6F2*, *MKX*), protein phosphorylation (*MAP4K3*, *PRKD2*, *ROR1*, *STK33*), positive regulation of the ERK1 and ERK2 cascade (*ROR1*, *PRKD2*), Wnt signaling (*ROR1*, *DACT3*), and cell adhesion (*PRKD2*, *SYMPK*).

### Gene-set enrichment analysis

MetaCore^TM^ gene-set enrichment analysis was performed using genes with p<0.01 in the trio (195 genes) or case-control (246 genes) based meta-analyses (Table F in S1 File). We identified two significantly enriched pathways (FDR-corrected p<0.05): dynein-dynactin motor complex in axonal transport in neurons (FDR-corrected p = 0.02), and chromosome condensation in prometaphase (FDR-corrected p = 0.02) (Table G in S1 File). In addition, we identified 111 significantly enriched (FDR-corrected p<0.05) non-redundant (REVIGO-clustered) GO processes (Table H in S1 File). GO term clusters included processes relevant to heart defects including cellular response to hormone stimulus, angiogenesis and biological adhesion.

### Gene annotations and prioritization

Of the 38 genes with suggestive evidence of association (p<1E-3) in either the family-based or case-control meta-analysis, 19 protein-coding and 2 RNA genes had a lower meta-analysis p-value than the p-values in contributing datasets i.e. the evidence for association was stronger in the combined data than in either individual dataset (Table 5). For the 21 genes with meta-analysis p-value less than the individual study p-values, the majority (95%) of variants included in the analyses were intronic (Table I in S1 File). Heart expression data from E9.5 and E14.5 mouse embryos [18] were available for 15 of the 19 protein-coding genes, of which eight (53%) were in the top quartile of expression at one or both time points (Table 5). We propose these eight genes (*ARF5*, *EIF4E*, *KPNA1*, *MAP4K3*, *MBNL1*, *NCAPG*, *NDFUS1*, *PSMG3*) as CTD candidate genes.

**Table 5:** Genes with suggestive evidence of association (p<1E-03) in either dataset and with a meta-analysis p-value that is lower than that obtained in either of the contributing analyses.

Gene	Gene Name	Function	Gene-based Test	Dataset 1 p-value^a^	Dataset 2 p-value^a^	Meta-analysis p-value	Day 9.5^b^	Day 14.5^b^
***ARF5***	ADP-ribosylation factor 5	Protein-coding	SKAT-C	3.84E-03	2.17E-02	8.67E-04	95.3	88.8
***CHODL***	Chondrolectin	Protein-coding	SKAT-C	2.80E-02	1.32E-03	4.15E-04	5.8	10.4
***DCAF16***	DDB1 and CUL4 associated factor 16	Protein-coding	SKAT-C	1.70E-03	3.98E-02	7.18E-04	No data	No data
***EIF4E***	Eukaryotic translation initiation factor 4E	Protein-coding	SKAT-C	2.55E-02	1.24E-03	3.60E-04	81.7	76.4
***FAM225A***	Family with sequence similarity 225 member 1	RNA gene	SKAT-C	3.86E-05	5.28E-02	2.87E-05	--	--
***FBXO47***	F-box only protein 47	Protein-coding	SKAT-C	2.17E-03	3.69E-02	8.36E-04	43.8	28.3
***GLIPR1***	Glioma pathogenesis-related protein 1	Protein-coding	eFBAT-MM	2.85E-02	2.88E-03	8.55E-04	57.6	34.9
***KPNA1***	Karyopherin alpha-1	Protein-coding	eFBAT-MM	5.37E-02	1.50E-03	8.41E-04	82.4	77.8
***LEXM (C1orf177)***	Chromosome 1 open reading frame 177	Protein-coding	SKAT-C	2.52E-04	4.19E-02	1.32E-04	41.4	16.1
***LINC00207***	Long intergenic non-protein coding RNA 207	RNA gene	eFBAT-MM	2.01E-02	2.86E-03	6.17E-04	--	--
***LOC100287036***	Uncharacterized LOC100287036	Protein-coding	SKAT-C	6.34E-03	2.49E-03	1.90E-04	No Data	No data
***MAP4K3***	Mitogen-activated protein kinase kinase kinase 3	Protein-coding	eFBAT-MM	1.56E-03	2.11E-02	3.74E-04	76.1	76.9
***MBNL1***	Muscleblind-like splicing regulator 1	Protein-coding	eFBAT-MM	3.29E-02	3.70E-04	1.50E-04	66.9	78.2
***NCAPG***	Non-SMC condensin 1 complex subunit G	Protein-coding	SKAT-C	6.79E-04	5.54E-02	4.21E-04	83.5	70.5
***NDUFS1***	NADH-ubiquinone oxidoreductase Fe-S protein 1	Protein-coding	SKAT-C	8.63E-02	8.79E-04	7.95E-04	94	97.1
***PRKD2***	Protein kinase D2	Protein-coding	SKAT-C	1.17E-03	2.31E-02	3.12E-04	48.1	66.9
***PSMG3***	Proteasome assembly chaperone 3	Protein-coding	SKAT-C	6.50E-02	2.55E-05	2.37E-05	79	54.1
***RGS16***	Regulator of G protein signaling	Protein-coding	SKAT-C	5.31E-03	6.65E-03	3.97E-04	56.4	38.9
***ROR1***	Receeptor tyrosine kinase-like orphan receptor 1	Protein-coding	eFBAT-MM	2.42E-02	2.02E-03	5.33E-04	65.6	63.5
***SHD***	SH2 domain-containing protein D	Protein-coding	SKAT-C	6.86E-04	1.82E-02	1.53E-04	65.5	36.2
***SIGLEC11***	Sialic acid-binding immunoglobulin-like lectin 11	Protein-coding	eFBAT-MM	4.09E-03	3.10E-03	1.56E-04	36.2	No data

^a^ When meta-analysis with p<1E-03 is FBAT, dataset 1 = CHOP Trios and dataset 2 is PCGC Trios. When meta-analysis with p<1E-03 is SKAT, dataset 1 = CHOP-CC1 patients with a CTD and controls and dataset 2 is CHOP-CC2 patients with a CTD and controls.

^b^ Heart expression percentile rank [18].

## Discussion

Our comprehensive genome-wide, gene-based analysis of common and rare variants identified 38 genes with suggestive evidence of association (meta-p<1E-3) with CTDs, as well as relevant biological pathways and processes that were significantly enriched (FDR-corrected p<0.05) among the genes with the most significant p-values in gene-based analyses. Based on both statistical evidence (i.e. the evidence for association was stronger in the meta-analysis than in any of the contributing studies) and gene expression data (top quartile of expression in mouse heart at E9.5 or E14.5) we propose eight genes (*ARF5*, *EIF4E*, *KPNA1*, *MAP4K3*, *MBNL1*, *NCAPG*, *NDFUS1*, *PSMG3*) as CTD candidate genes.

Four of the CTD candidate genes suggested by our work have not been associated with normal or abnormal heart development. These four genes are: ADP ribosylation factor 5 (*ARF5*), which encodes a GTP-binding protein involved in protein trafficking; karyopherin subunit alpha 1 (*KPNA1*), which functions in nuclear protein import; NADH:Ubiquinone oxidoreductase core subunit protein coding S1 (*NDUFS1*), which encodes the core subunit of the mitochondrial membrane respiratory chain NADH dehydrogenase, and; proteasome assembly chaperone 3 (*PSMG3*), which encodes a chaperone protein.

The known function of the remaining four candidate genes suggests that their altered expression could cause CHDs. Of these genes, the most significant association was observed for muscleblind-like splicing regulator 1 (*MBNL1*, eFBAT-MM meta-p = 1.5E-04). This gene encodes a CH3-type zinc finger protein (MBNL1) that is a key regulator of pre-RNA alternative splicing. Evidence that splicing regulators contribute to the etiology of CHDs is provided by the identification of a genome-wide, significant excess of damaging *de novo* and loss-of-function heterozygous mutations in another key splicing regulator, *RBFOX2*, in patients with CHDs [17].

Several additional lines of evidence also support a role for *MBNL1* in cell differentiation and heart development. For example, *MBNL1* and *RBFOX2* appear to co-regulate the splicing changes that lead to the differentiation of pluripotent stem cells [42]. In addition, in the nucleotide repeat expansion disorder, myotonic dystrophy, reduced *MBNL1* splicing activity (due to binding of MBNL1 protein to the expansion RNA) is thought to play a major role in determining the disease phenotype, which includes several cardiovascular abnormalities (conduction defects, arrhythmias, mitral valve prolapse) [43, 44]. There is also evidence that *MBNL1* is involved in the fetal to adult transition in alternative splicing patterns in the heart [45], and that *MBNL1* negatively regulates TGF-β signaling and the epithelial-mesenchymal transition in the endocardial cushions by restricting the timing and amount of TGF- β production in the atrioventricular canal and outflow tract endocardium [46, 47]. Mice null for MBNL1 protein present with abnormal heart valve development, regurgitation across both the in- and outflow valves, and ostium secundum septal defects [47].

Further evidence that genes involved in RNA splicing may be associated with CTDs is provided by our gene-set enrichment analyses. Specifically, genes mapping to the GO process ‘regulation of RNA splicing’ (GO:0043484) were significantly enriched (FDR-adjusted p = 0.03) among genes with association p<0.01 in our meta-analyses. In addition to *MBNL1*, seven genes mapping to this process (*CLK3*, *DDX5*, *JMJD6*, *SRSF2*, *SRSF9* and *TMBIM6*) had meta-analysis p<0.01 (meta-analysis p-value range: 2E-03 to 8E-03) in either the family-based (i.e. eFBAT-MM) or case-control (i.e. SKAT-C) meta-analysis.

The second most significant of our proposed CTD candidate genes was eukaryotic translation initiation factor 4D (*EIF4E*, SKAT meta-p = 3.6E-04). The encoded protein, eIF4F, directs ribosomes to the mRNA 5’-cap and is a key factor in initiation of translation of many mRNAs [48]. Zhang et al. have presented evidence that eIF4E is involved in heart development via the p53-Rbm24 loop [49]. Specifically, they demonstrated that the multifunctional RNA-binding protein, Rbm24, prevents binding of eIF4E to p53 RNA, thereby repressing p53 translation and p53-dependent apoptosis. Further, they showed that mice deficient for *Rbm24* develop endocardial cushion defects as a result of aberrant binding of eIF4E to p53 RNA resulting in overexpression of p53. Mutations in *EIF4E* have also been implicated as a cause of autism in humans [50], and enhanced eIF4E activity has been associated with autism-like phenotypes in animal models [51]. Hence, our finding adds *EIF4E* to the growing list of genes that may be related to both CHDs and neurodevelopment disabilities such as autism [17, 52].

Our study also identified the mitogen-activated protein kinase kinase kinase kinase 3 (*MAP4K3*) as a CTD candidate gene. The product of this gene is an upstream activator of the c-Jun-N-terminal kinase (JNK) signal transduction pathway, which is involved in several processes relevant to heart development (e.g. cell growth, differentiation and survival, apoptosis) [53]. Downstream effectors of JNK signaling relevant to heart development include the tumor suppressor/apoptosis gene, *p53* (discussed above), and *SMAD4*. In a mouse model, disruption of *Smad4* in neural crest cells resulted in multiple malformations including defects of the outflow tracts and ventricles [54]. Further, in humans, *SMAD4* gain of function mutations cause Myhre syndrome, which includes CHD as a common (~2/3rds of patients) phenotypic finding [55]. There is also evidence that *MAP4K3* is a central regulator of autophagy, a process that is critical for maintaining the supply of free amino acids for protein synthesis [56] that is required for embryonic growth and development.

Finally, our analyses identified non-SMC condensing I complex subunit G (*NCAPG*, SKAT meta-p = 4.2E-04) as a CTD candidate gene. The protein encoded by this gene forms part of the condensin complex, which is involved in mitotic chromatin condensation [57]. Further, our analyses indicated that genes in the MetaCore pathway map, “Chromosome condensation in prometaphase) were also significantly enriched (FDR-adjusted p = 0.02) among genes with association p<0.01 in our meta-analyses. In addition to *NCAPG*, a second member of the condensing complex, *NCAPH* (FBAT meta-p = 9.13E-03), and *BAZ1B* (FBAT meta-p = 5.62E-03), which is part of the WICH chromatin remodeling complex, had meta-analysis p<0.01. The involvement of chromatin-related genes, particularly H3K4me-H3K27me pathway genes, in CHD etiology has been suggested by studies of *de novo* mutations [18]. Our findings suggest that other classes of chromatin-modifiers may also contribute to CHDs.

We have previously conducted SNP-level (MAF≥0.05) GWAS using the same datasets as in the current gene-based analyses [20]. In our meta-analysis of the SNP-level results, we identified 36 variants with suggestive evidence of association (P≤1E-5). However, no association was genome-wide significant (P<5E-8). Further, none of the SNPs with suggestive evidence of association were located in, or within 1kb up or downstream of, the genes with suggestive evidence of association (Table 5) in the current, gene-based analyses. However, it should be noted that the SNP-level analyses were restricted to include only common variants and used slightly different configurations of the data. Specifically, in the SNP-level analyses we compared the cases used in CHOP-CC2 to all available CHOP pediatric controls (N = 2,976 controls), and the SNP-level meta-analysis was based on the combined results from CHOP-Trios, PCGC-Trios and the case-control analyses.

To our knowledge, this is the first gene-based genome-wide analysis of CTDs that is based on data for both common and rare variants. Because the various gene-based approaches have different underlying assumptions, strengths and limitations, we used two different gene-based approaches, eFBAT-MM and SKAT-C, to optimize the probability of identifying CTD-related genes. The family-based approach, eFBAT-MM, is robust to population stratification bias, but assumes that all variants within a gene have effects in the same direction and that the effect size is inversely proportional to the MAF. In contrast, the case-control approach, SKAT-C, is subject to stratification bias, but does not make assumptions about the direction of association. Therefore, SKAT-C is more powerful than eFBAT-MM when a large proportion of protective and neutral variants are present in a gene, and the converse is true when this proportion is small. Given the differences between the two methods, the lack of overlap in the genes identified by the two approaches is not particularly surprising.

Although the gene-based approaches used in our analyses had a lower multiple-testing burden than SNP-based GWAS, the criterion for achieving statistical significance (corrected p~2.5E-06) remained quite stringent. This, in combination with our relatively small sample sizes, suggests that associations with true CTD-related genes may have been missed in our analyses due to low study power. Further, the Q-Q plots for the eFBAT-MM analysis of individual datasets (Fig 2) indicate that this test may be too conservative, which would have also negatively impacted our power to detect a true association. Given these considerations, genes with suggestive evidence of association (meta-p<1E-03) and pathways and processes with FDR p<0.05 appear to be strong targets for further investigations of the genetic basis of CTDs.

In our analyses, we combined data across different CTD phenotypes, which could have obscured associations if the etiology of the individual phenotypes is distinct. For example, mutations in laterality genes (e.g. *CFC1*, *FOXH1*) have been observed in association with TGA [58, 59], suggesting that at least some cases of TGA might be more appropriately classified as laterality defects rather than CTDs. However, the preponderance of evidence suggests that the various CTD phenotypes share common genetic underpinnings. Studies of familial recurrence patterns, phenotypes of patients with known genetic syndromes (e.g. 22q11.2 deletion syndrome) and studies in animal models all indicate that the various CTD phenotypes share genetic risk factors. Moreover, studies of rare *de novo* and inherited variants in humans provide evidence that the genes involved in CHDs may be shared across even broader categories of defects. For example, Jin et al. reported genome-wide significant excess of damaging *de novo* and loss-of-function heterozygous mutations in seven genes among 2,871 patients with CHD. Of these seven genes, mutations in six were observed across broad CHD categories (i.e. CTDs, left-sided lesions and/or other CHDs) [17]. Hence, while studies of CTDs as a group might miss phenotype-specific associations, such studies appear to be appropriate for genes that contribute broadly to CHD risk and for genes that influence the spectrum of CTDs.

In summary, our genome-wide, gene-based analyses of common and rare variants identified enriched pathways and biological processes and candidate genes for CTDs. Our findings provide evidence for new CTD-related candidate genes, as well as support and expand on prior evidence implicating chromatin-related genes and splicing-regulators as determinants of CHD risk.

## Conclusions

To our knowledge, this is the first study reporting the results of gene-based, genome-wide association studies for CTDs. The results of our study provide evidence for eight CTD candidate genes, of which four have previously been implicated in heart development and four are novel candidates. Thus, these findings add to our understanding of the complex, genetic etiology of CTDs, which may, in turn, enhance our ability to understand, predict and ultimately improve clinical outcomes for this patient population.

## Supporting information

S1 File The file includes author group details of Pediatric Cardiac Genomics Consortium (PCGC) and complete results from genome-wide gene-level analyses, meta-analyses, and gene-set enrichment analyses. (XLSX)

S1 Fig Quantile-quantile plot of eFBAT-MM test gene-level p-values in CHOP Trios. (TIF)

S2 Fig Quantile-quantile plot of eFBAT-MM test gene-level p-values in PCGC Trios. (TIF)

S3 Fig Quantile-quantile plot of SKAT-C test gene-level p-values in CHOP-CC1 cohort. (TIF)

S4 Fig Quantile-quantile plot of SKAT-C test gene-level p-values in CHOP-CC2 cohort. (TIF)
