# Supplementary figures and images for: Gene-based genome-wide association studies and meta-analyses of conotruncal heart defects
Source: PLoS One. 2019 Jul 17;14(7):e0219926. doi: 10.1371/journal.pone.0219926 (PMC6636758; doi:10.1371/journal.pone.0219926)

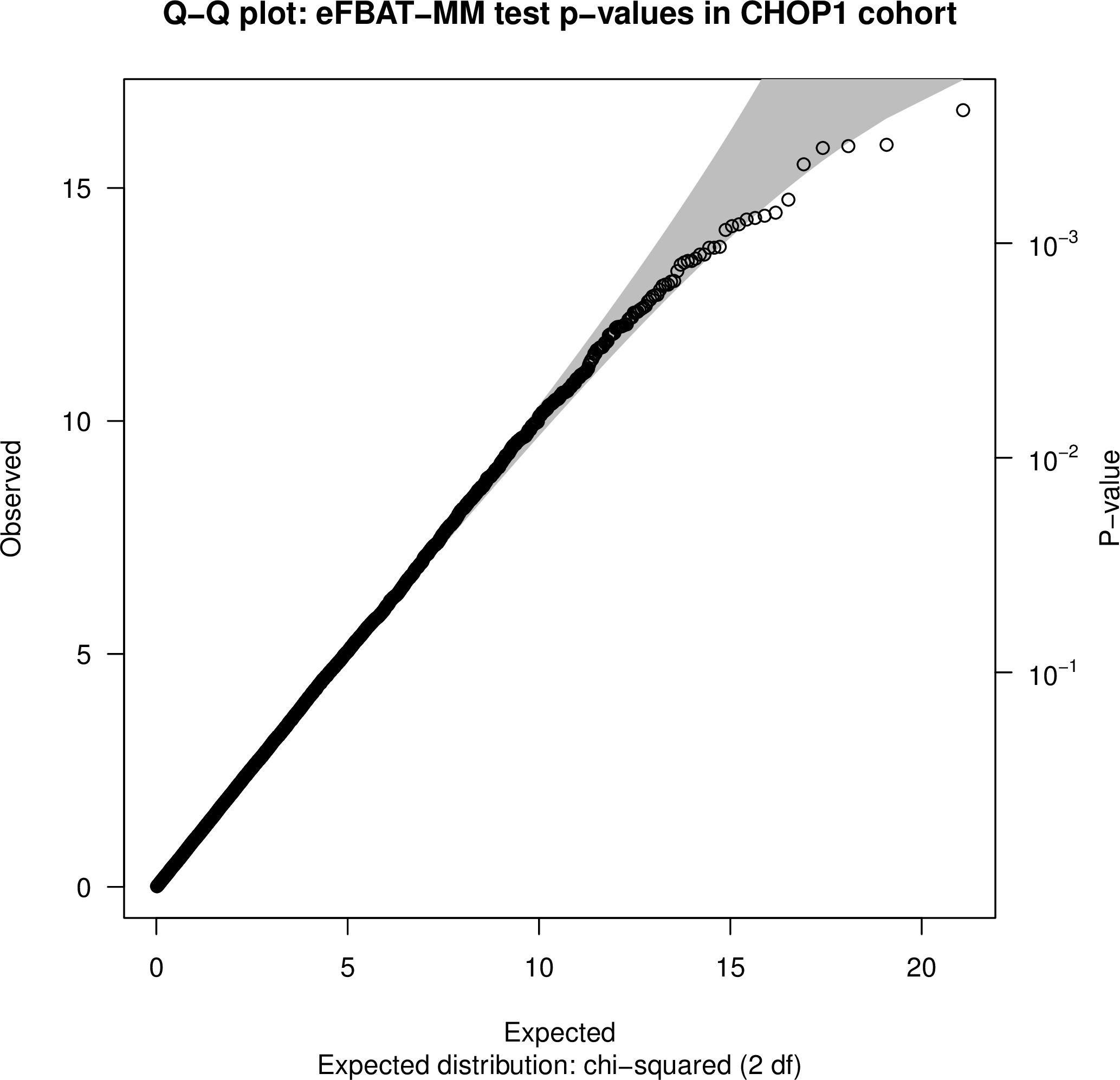

Supplement: S1 Fig — (TIF) [file pone.0219926.s002.tif]

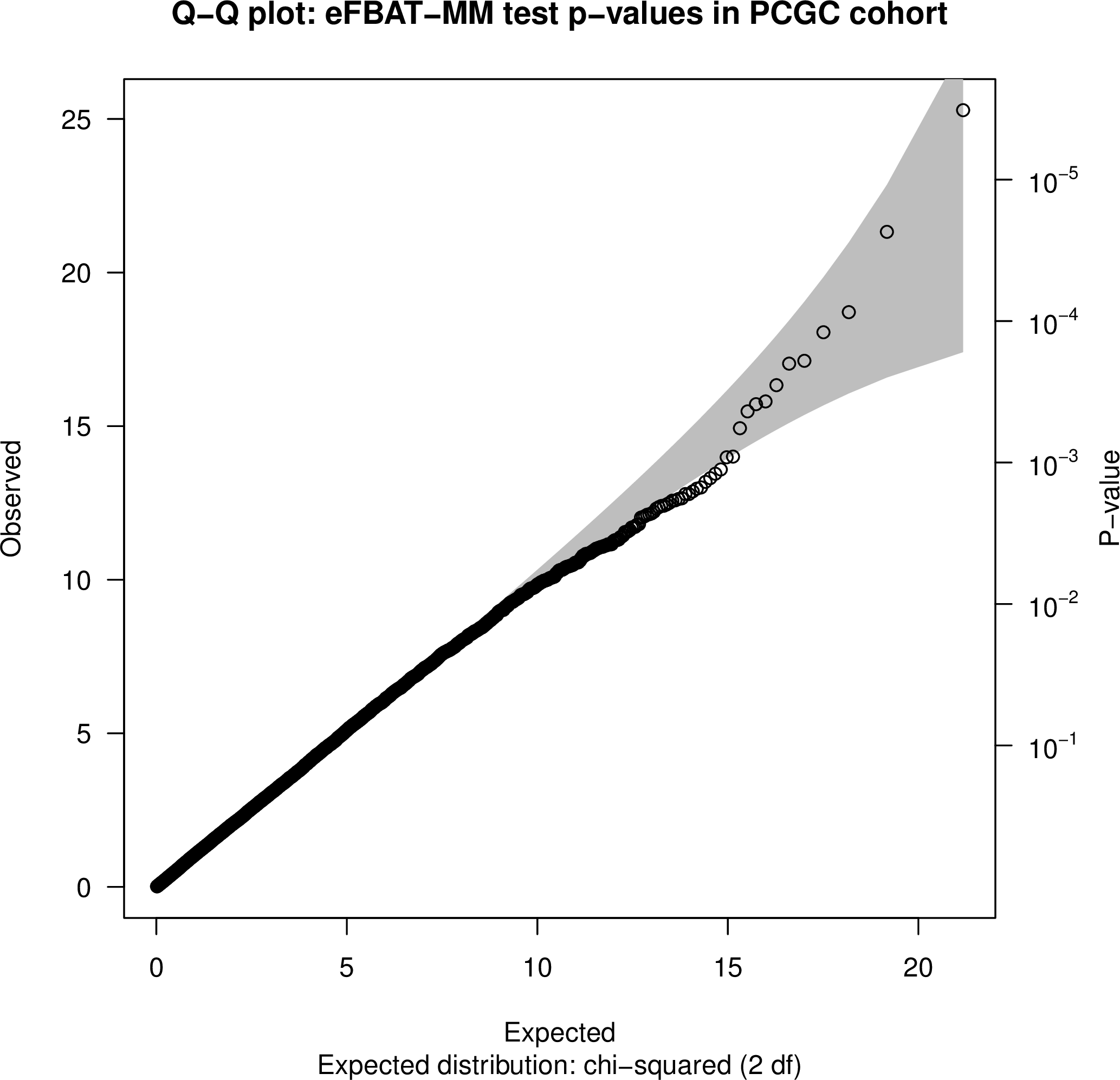

Supplement: S2 Fig — (TIF) [file pone.0219926.s003.tif]

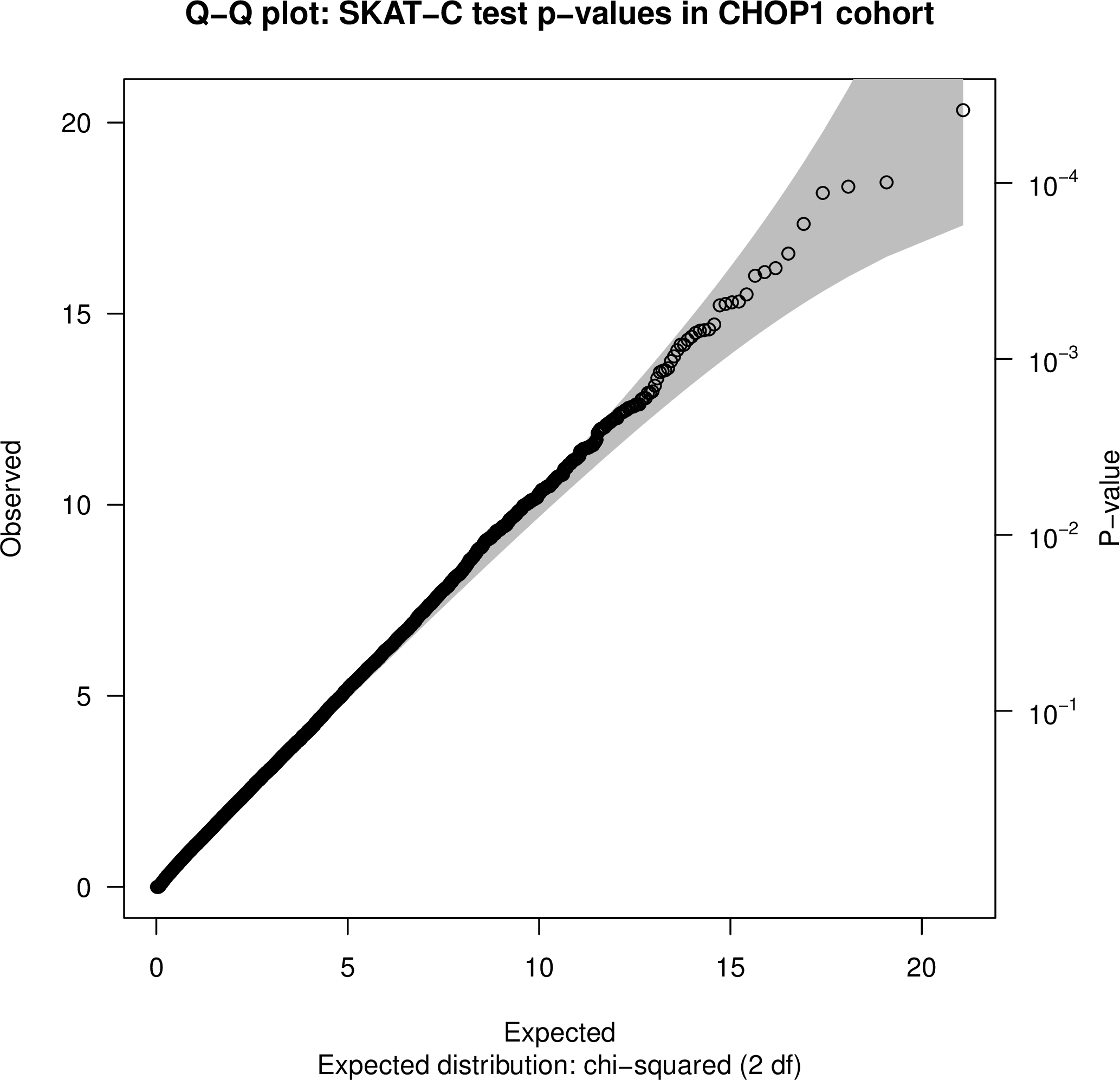

Supplement: S3 Fig — (TIF) [file pone.0219926.s004.tif]

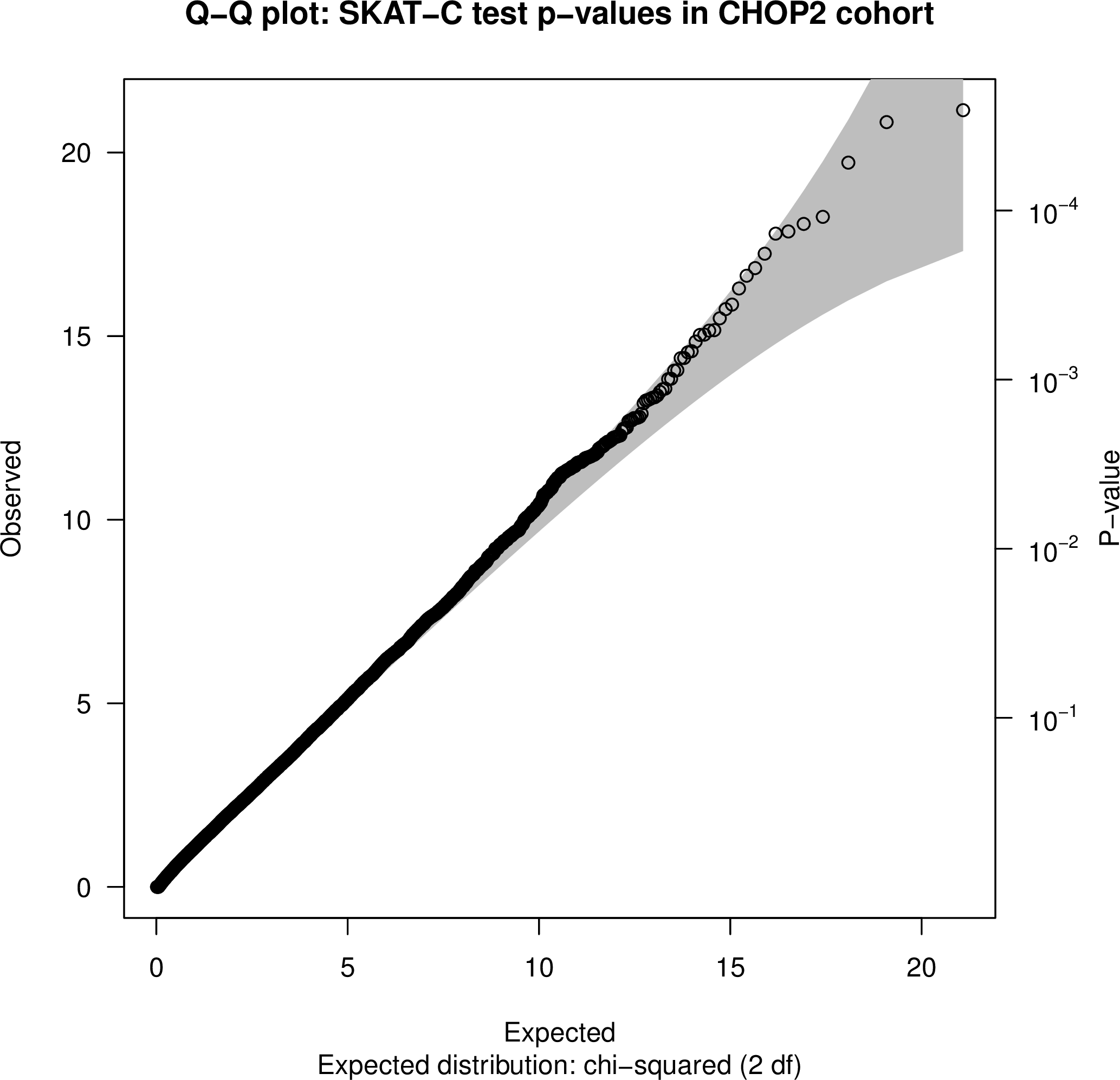

Supplement: S4 Fig — (TIF) [file pone.0219926.s005.tif]
